# Optimization of SPECT-CT Hybrid Imaging Using Iterative Image Reconstruction for Low-Dose CT: A Phantom Study

**DOI:** 10.1371/journal.pone.0138658

**Published:** 2015-09-21

**Authors:** Oliver S. Grosser, Dennis Kupitz, Juri Ruf, Damian Czuczwara, Ingo G. Steffen, Christian Furth, Markus Thormann, David Loewenthal, Jens Ricke, Holger Amthauer

**Affiliations:** Department of Radiology and Nuclear Medicine, University Hospital Magdeburg, Magdeburg, Germany; Chongqing University, CHINA

## Abstract

**Background:**

Hybrid imaging combines nuclear medicine imaging such as single photon emission computed tomography (SPECT) or positron emission tomography (PET) with computed tomography (CT). Through this hybrid design, scanned patients accumulate radiation exposure from both applications. Imaging modalities have been the subject of long-term optimization efforts, focusing on diagnostic applications. It was the aim of this study to investigate the influence of an iterative CT image reconstruction algorithm (ASIR) on the image quality of the low-dose CT images.

**Methodology/Principal Findings:**

Examinations were performed with a SPECT-CT scanner with standardized CT and SPECT-phantom geometries and CT protocols with systematically reduced X-ray tube currents. Analyses included image quality with respect to photon flux. Results were compared to the standard FBP reconstructed images. The general impact of the CT-based attenuation maps used during SPECT reconstruction was examined for two SPECT phantoms. Using ASIR for image reconstructions, image noise was reduced compared to FBP reconstructions for the same X-ray tube current. The Hounsfield unit (HU) values reconstructed by ASIR were correlated to the FBP HU values(R^2^ ≥ 0.88) and the contrast-to-noise ratio (CNR) was improved by ASIR. However, for a phantom with increased attenuation, the HU values shifted for low X-ray tube currents I ≤ 60 mA (p ≤ 0.04). In addition, the shift of the HU values was observed within the attenuation corrected SPECT images for very low X-ray tube currents (I ≤ 20 mA, p ≤ 0.001).

**Conclusion/Significance:**

In general, the decrease in X-ray tube current up to 30 mA in combination with ASIR led to a reduction of CT-related radiation exposure without a significant decrease in image quality.

## Introduction

In the early 2000s the introduction of hybrid imaging techniques combining positron emission tomography (PET) or single photon emission computed tomography (SPECT) with X-ray computed tomography (CT) for diagnostic imaging represented a significant improvement over stand-alone applications of individual imaging modalities [1,2]. As an intrinsic part of the hybrid imaging device, the CT permits accurate anatomic localization of tracer uptake and can be used for CT-based attenuation correction (CTAC) of emission data [3,4].

As the two examinations are performed within a close time frame, imaging artifacts induced by patient movements or due to different filled organs (e.g. stomach, bladder) can be reduced or even avoided. Over the period of development of hybrid imaging, there have also been great improvements in diagnostic CT applications [5].

However, the introduction of new CT protocols (e.g. cardiac imaging) and the trend towards replacement of conventional X-ray imaging by CT examinations [6] have markedly increased medical radiation exposure and the risk of radiation-induced neoplasia in the population [5]. Worldwide, CT imaging in medical applications is responsible for more than 40% of the accumulated effective dose [7]. Studies suggest that, up to the age of 75, 0.6–3.2% of the cumulative risk of cancer can be attributed to radiation exposure caused by diagnostic imaging in developed countries [8,9]. Therefore, medical CT imaging needs to be optimized.

In hybrid SPECT-CT imaging, the CT component can be used for (a) diagnostic CT applications (if the CT component has the appropriate imaging capabilities), (b) localization diagnostics by low-dose CT (LD-CT), or (c) exclusively for CTAC of emission data [10]. However, even with currently used CT protocols, a significant exposure is accumulated. Effective CT exposures of 7.0 ± 3.3 mSv (range = 3.7–11.1 mSv) were reported for consecutive SPECT-CT examinations with ^111^In-octreotide in tumor diagnostics [11]. The same authors reported a CT exposure of 3.8 ± 3.9 mSv (range = 0.2–12.4 mSv) for LD-CT imaging of bone metabolism with ^99m^Tc-methylene diphosphonate [11]. Both radiation risk and protection of patients in clinical SPECT-CT are currently controversial issues [12]. To minimize radiation exposure in diagnostic CT technical innovations (e.g. angular modulation of the tube current) and new reconstruction technologies (e.g. iterative CT reconstructions) were established [6,13,14].

However, such innovative techniques have to be optimized for LD-CT applications. Such an optimization is usually a multifactorial process of adjustment of X-ray tube current, X-ray tube voltage, pitch and image reconstruction (e.g. processing filters) to the clinical need and the condition of the patient. In general, the optimization process is a trade-off between image quality and radiation exposure. Other workers have reported their experience gathered with LD-CT applications in contrast-enhanced CT angiography (CCTA) [15,16], but this imaging is performed under considerably different conditions and objectives. Furthermore, there are several studies presenting results of the long-term process of examining the influence of (LD-CT or) CT in PET imaging [17–19]. However, the impact of iterative reconstruction algorithms in LD-CT hybrid imaging and the effect of dose reduction on the CTAC of SPECT emission data have not yet been fully evaluated.

It was the purpose of this study to investigate the influence of iterative CT image reconstruction algorithms on CT image quality (e.g. image noise, contrast-to-noise ratio) and on CTAC SPECT-images using LD-CT protocols. The examinations were performed with a focus on phantom geometries representing different adult patients.

## Materials and Methods

### SPECT-CT

All examinations were performed with a dedicated hybrid SPECT-CT (Discovery NM/CT 670, GE Healthcare, Milwaukee, USA). The integrated CT component is identical in construction to a 16-slice-CT used in diagnostic CT imaging (model: Bright Speed 16, GE Healthcare, Milwaukee, USA). The standard CT reconstruction was performed by filtered back projection (FBP). Additionally, the system includes an iterative CT reconstruction algorithm (ASIR, Adaptive Statistical Iterative Reconstruction, GE Healthcare, Milwaukee, USA) established in diagnostic CT imaging [14,20–22]. ASIR uses the images reconstructed by FBP as starting information. The parameter ASIR-level (from 0% to 100% selectable in increments of 10%) defined the merging of the iterative reconstruction and the FBP reconstruction. The imaging protocol was chosen in accordance with the basic scan protocol for LD-CT in SPECT-CT imaging in routine clinical practice. Imaging and reconstruction protocols are defined below.

### Low-dose CT Imaging and CT Phantom

LD-CT phantom measurements were performed with a standardized phantom set (Catphan 500^®^, The Phantom Laboratory, Salem, NY, USA). Scattering and attenuation of the phantom corresponded to the head of an adult patient or to the abdominal region of a lean patient (outside-diameter = 210 mm; e.g. children), henceforth referred to as head geometry. To increase attenuation and scattering, comparable to the body/abdominal region of an adult, the phantom was extended by an additional annulus with a water equivalent attenuation (outside diameter = 350 mm; model: CTP540, The Phantom Laboratory, Salem, NY, USA), henceforth referred to as body geometry. Both geometries represented standardized set-ups for performance tests in diagnostic CT.

CT scans of the phantom were performed in an axial field-of-view (FOV) with a diameter of 50 cm by helical scans with a gantry rotation time t_rot_ = 0.8 s, a table pitch p = 1.375 and a primary collimation of 16 x 1.25 mm. The LD-CT scans were performed without an angular variation of the X-ray tube current. The X-ray tube voltage was set to a fixed value (U = 120 kVp) in accordance with the manufacturer’s setting for CTAC. Scans were performed with different tube currents I = 10, 20, 30, 40, 50, 60, 80, 100, 120 mA to estimate the low-dose performance of the system for different CT dose exposures. The CT dose exposures for both phantom geometries were estimated for all X-ray tube currents by a calibrated dose meter (electrometer, DIADOS^®^ with CT adapter and ionization chamber, PTW Freiburg, Freiburg, Germany).

Image reconstruction was performed in accordance with the requirements of a clinically applied LD-CT protocol defined for SPECT-LD-CT imaging by the manufacturer. CT images were reconstructed in slices 3.75 mm thick (512 x 512 matrix, 0.977 x 0.977 mm) with FBP (with convolution kernel = ‘standard’; particularly used for routine examinations like abdomen and pelvis scan in diagnostic CT; recommended by the manufacturer for LD-CT) and additionally with the ASIR algorithm. The ASIR-levels selected to estimate the basic potential of the iterative reconstruction algorithm were ASIR = 0% (equivalent to FBP reconstruction), ASIR = 50% (ASIR50%) and ASIR = 100% (ASIR100%).

**Fig 1:**
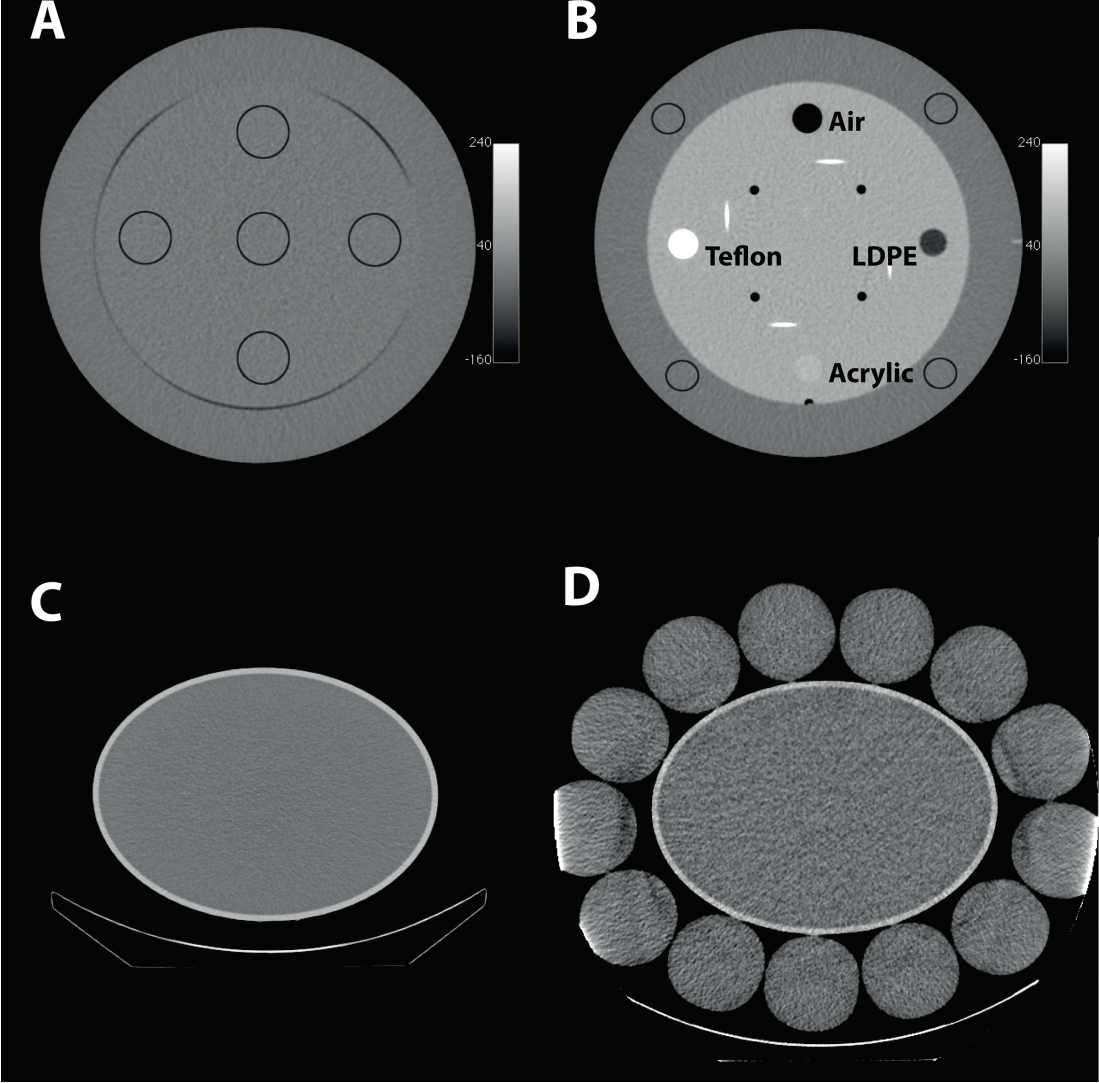
Phantom geometries. (A) Central transaxial slice across the uniformity module of the Catphan^®^ 500 phantom (outer diameter 200 mm) with 5 ROIs, (B) Catphan^®^ 500 phantom with 4 different labeled sensitometry samples and the positions of the background ROIs in the outer part of the phantom section. SPECT examinations were performed with (C) an elliptic phantom (semi-axes: 310 x 230 mm) and (D) the same elliptic phantom extended by additional bottles. CT phantom geometry (A, B) and SPECT phantom geometries (C, D) were not represented using the same scale.

Performance parameters were determined by regions of interest (ROIs) analysis. Image noise was estimated from ROIs in the uniformity module (section model: CTP486) of the Catphan^®^ phantom. These ROIs (n = 5) were always positioned in the same axial slice as presented in Fig 1A. Image noise was defined by the standard deviation of the pixel values. The mean reconstructed HU value (mean_high_) was estimated from ROI analysis of the high contrast module of the Catphan^®^ phantom (section model: CTP401, Fig 1B). The measurements were performed for 4 sensitometry samples made of Acrylic, Teflon, low-density-polyethylene (LDPE) and air. The contrast-to-noise ratio (CNR) for each module was calculated in relation to the background region by

$$CNR=\frac{mean_{high}-mean_{BKG}}{SD_{BKG}}$$ (1)

where mean_BKG_ corresponded to the mean HU value in the background-ROI and SD_BKG_ corresponded to the standard deviation of the background-ROI. The background region was defined by 4 ROIs in the outer ring of the high contrast module (Fig 1B).

### SPECT-CT Imaging and SPECT-CT Phantom

**Fig 2:**
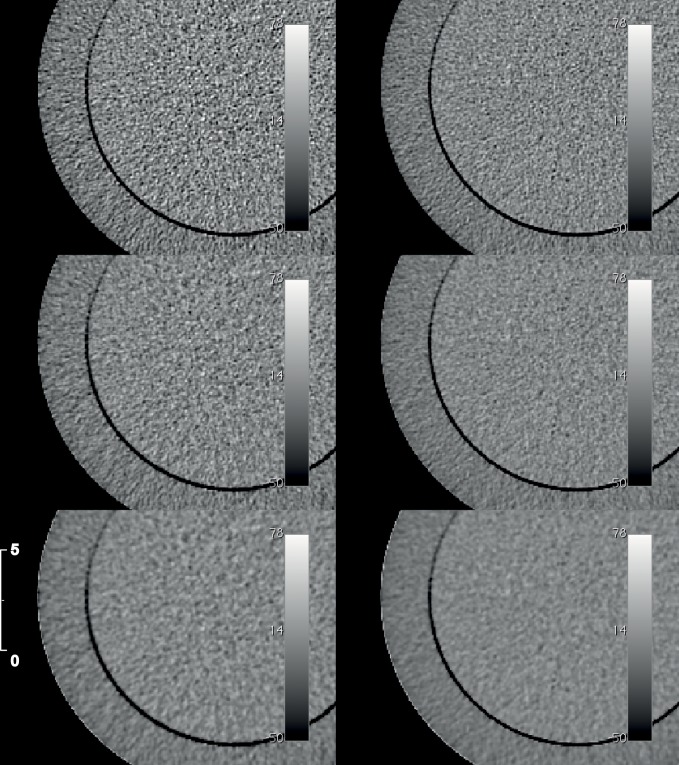
Reconstructed detail of the uniformity module of the Catphan^®^ 500 phantom. The phantom was scanned with a constant tube voltage (U = 120 kVp) and with two different X-ray tube currents (left: I = 20 mA; right: I = 40 mA). The images were reconstructed by FBP, ASIR50% and ASIR100% (top row: FBP, middle row: ASIR50%, bottom row: ASIR100%). All images were windowed with the same window level and width. The scale is in centimeters.

The influence of the LD-CT image quality on SPECT imaging was examined by measurements using a standard elliptical SPECT phantom (Lung-Spine SPECT Phantom without modules, Biodex Medical Systems, Shirley, NY, USA). SPECT data were obtained for both: (1) the elliptical phantom (semi-axes: 310 x 230 mm, height: 190 mm; Fig 1C) and (2) the same elliptical phantom surrounded by 13 plastic bottles (diameter = 87 mm each) filled with purified water (Fig 1D) to increase attenuation and scattering.

The elliptical phantom was homogeneously filled with 550 MBq ^99m^Tc-pertechnetate diluted in water. SPECT imaging was performed over 360° (140 keV ± 10%, 60 projections at steps of 6°and 30 sec/projection) for a single bed position. A separate scatter window was measured at 120 keV ± 5%. The phantom-to-detector distance was minimized by the real-time automatic body contouring of the gamma camera. Several LD-CT scans were executed for CTAC of the SPECT data. The CT scan protocol, the variation of the X-ray tube currents and the image reconstruction of the CT images were performed as previously described for CT examinations with the Catphan^®^ phantom. The LD-CT images, which were used for attenuation correction, were reconstructed by FBP (filter: standard), ASIR50% and ASIR100%. Data processing was carried out on a dedicated workstation (Xeleris 3^®^, GE Healthcare, Milwaukee, USA). The attenuation map (μ map) was calculated by a system-specific algorithm [23]. The SPECT image reconstruction was performed by an iterative algorithm (3D-OSEM: 3D-Ordered Subset Expectation Maximization) including resolution recovery, scatter correction and attenuation correction by μ maps estimated from LD-CT (Evolution^®^ Package, GE Medical, Milwaukee, USA).

The LD-CT, the μ map and the CTAC SPECT images were analyzed for all voxels of the ROIs (square 5 x 5 cm), which were positioned in the center of the elliptical phantom. The mean attenuation coefficient μ within the μ maps and the mean reconstructed counts within the SPECT images were determined for both SPECT phantom geometries.

### Radiation Dose

For the estimation of radiation exposure of LD-CT the CT dose index (CTDI_vol_) was determined for four different X-ray tube currents (I = 10, 40, 80, and 120 mA). The CTDI_vol_ values were automatically documented in a dose report by the SPECT-CT. The documented values were validated by measurements with the standardized CTDI_vol_ phantom and a calibrated dose meter. Furthermore, the effective dose resulting from these scan protocols was calculated with the software package CT-Expo™ [24] for abdominal examinations of normal adult patients (male/female). The calculation was performed in accordance with the ICRP publication 103 [25]. The length of the CT scan (l = 40 cm) covered the full field of view of a SPECT examination (CT-Expo^TM^, z-coordinates = 0–40).

### Statistical analysis

Data analyses were carried out using the software package R 2.15.3 (Foundation for Statistical Computing, Vienna, Austria, 2012, http://www.R-project.org). Descriptive parameters are given as mean and standard deviation as well as median and interquartile range (IQR; 25th-75th percentiles). HU values, Noise, CNR and reconstructed SPECT values were tested for normality by the Shapiro-Wilk test for every X-ray tube current. The dependencies between the X-ray tube current, image noise and CNR were analyzed by regression analyses. The level of agreement was estimated by Bland-Altman analysis [26]. Differences in image noise and CNR between reconstruction algorithms were analyzed using the Friedman test for paired data and t-test. All tests were performed two-sided and a p-value of < 0.05 was considered as statistically significant. The influence of different X-ray tube currents and ASIR-levels on the SPECT reconstructions was evaluated by a general linear model (GLM).

## Results

### CT Imaging

#### Noise

**Fig 3:**
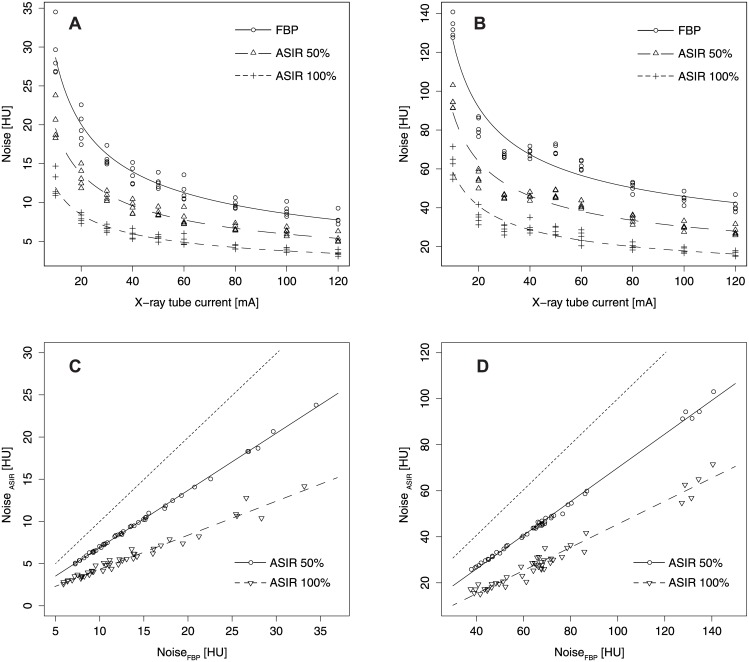
Image noise of FBP and iterative (ASIR) reconstructed CT images. (A, B) Image noise in the uniformity module of the Catphan^®^ 500 phantom reconstructed by the FBP, ASIR50% and ASIR100% versus X-ray tube current: (A) head geometry (Catphan^®^ phantom), (B) body geometry (Catphan^®^ phantom with additional annulus). (C, D) Scatterplots of image noise in ASIR (ASIR50% and ASIR100%) and FBP reconstructed images for equal X-ray tube currents: (C) head geometry, (D) body geometry. The line of identity for both methods (FBP and ASIR) is represented by the dotted line.

For equal scan conditions visual assessment revealed that the reconstructed images are less noisy with increasing ASIR-level than images reconstructed by FBP (Fig 2). In general, image noise increased with decreasing X-ray tube current for both FBP and ASIR. The ROI analysis of the image noise in the uniformity module of the Catphan^®^ phantom showed a 1/√I dependency for the head geometry (R^2^ = 0.95, p < 0.0001, Fig 3A) and for the body geometry (R^2^ = 0.97, p < 0.0001, Fig 3B). The noise in the images reconstructed with ASIR100% was significantly reduced compared to reconstructions with ASIR50% for both phantom geometries (p < 0.0001, Fig 3A and 3B). Compared to images reconstructed by FBP and for equal X-ray tube currents, the noise of ASIR50% images was reduced by a factor of 1.5 for the head geometry (1.4 for body geometry, Fig 3C) and for ASIR100% by a factor of 2.5 for the head geometry (2.0 for body geometry, Fig 3D). Images scanned with an X-ray tube current of I = 20 mA and reconstructed by ASIR50% showed an image noise, which was not significantly different from images scanned with I_Head_ = 40 mA—50 mA (I_Body_ = 80 mA) and reconstructed by FBP (p_Head_ ≥ 0.33, p_Body_ = 0.06, Fig 3A and 3B). For both geometries the noise level of images scanned with I = 20 mA and reconstructed by ASIR100% was significantly below the noise levels of images reconstructed by FBP and an X-ray tube current of I = 120 mA (both p = 0.04, Fig 3A–3D).

Image noise was normally distributed in both phantom geometries for all X-ray tube currents and reconstruction algorithms examined (p_Head_ > 0.43, p_Body_ > 0.4).

#### Hounsfield Unit (HU) Values, CT Bias

**Fig 4:**
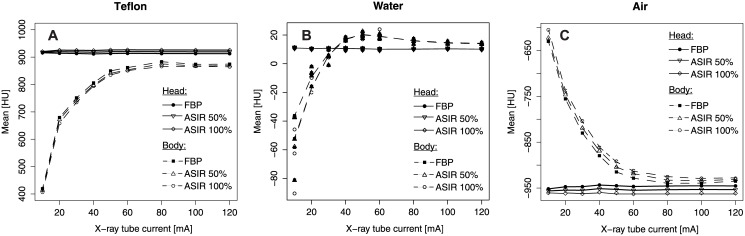
HU values of different sensitometry samples of the Catphan^®^ phantom. Mean HU versus X-ray tube current for FBP and ASIR reconstructions within three different sensitometry samples: (A) Teflon, (B) water equivalent and (C) air. All measurements were performed with a phantom for the head geometry (standard Catphan^®^ phantom) and for the body geometry (Catphan^®^ phantom with additional annulus). The tube voltage was always U = 120 kVp.

Regression analyses revealed high correlations of the mean HU values inside the ROIs in FBP and ASIR50% / ASIR100% reconstructed images for both phantom geometries (Table 1). The mean difference within the HU values and the level of agreement estimated by Bland-Altman analysis revealed a small deviation of the HU values reconstructed by FBP and ASIR50% or ASIR100% (Table 1). Fig 4 shows the dependence of the HU values on X-ray tube current for Teflon, water and air for both geometries. There was a systematic bias of HU values for the body geometry at low X-ray tube currents. The change in the HU values appeared for X-ray tube currents of I ≤ 60 mA (p ≤ 0.04) compared to the HU values estimated by CT scans with I = 120 mA. In contrast, a bias effect in HU values was not observed for the head geometry. The ASIR algorithm did not affect the HU values or the occurrence of a bias effect in HU values for decreased X-ray tube currents in LD-CT (Fig 4).

**Table 1:** Influence of the iterative reconstruction algorithm on the HU values. Comparison of ASIR 50% and ASIR 100% with FBP.

Material	ASIR-	R^2^	Mean difference in HU (FBP-ASIR)		Mean HU_FBP_ (I = 120 mA)
	Level	Head / Body	Head	Body	Head /Body
Acrylic	50	0.999 / 0.999	0.31 ± 0.32	0.28 ± 1.08	118.9 / 128.7
	100	0.999 / 0.993	0.63 ± 0.58	0.16 ± 10.02	
H_2_O	50	0.999 / 0.999	0.01 ± 0.27	0.00 ± 1.30	2.6 / 10.3
	100	0.988 / 0.881	0.02 ± 2.73	-0.09 ± 22.68	
LDPE	50	0.996 / 0.999	-1.30 ± 2.10	-2.01 ± 1.58	-86.8 / -73.6
	100	0.981 / 0.974	-2.59 ± 4.23	-4.17 ± 9.52	
Teflon	50	0.994 / 0.999	-6.01 ± 3.26	7.24 ± 4.08	912.8 / 873.4
	100	0.997 / 0.998	-12.00 ± 6.47	13.72 ± 15.32	
Uniform	50	0.999 / 1.000	0.01 ± 0.20	0.014 ± 0.57	10.1 / 14.2
	100	0.999 / 0.994	-0.01 ± 0.68	0.35 ± 7.42	
Air	50	0.878 / 0.999	7.25 ± 3.75	-8.09 ± 6.12	-945.3 / -934.9
	100	0.890 / 0.996	14.41 ± 7.64	-18.45 ± 16.94	

Correlation between the mean HU from LD-CT images reconstructed by ASIR compared to the standard algorithm (FBP). Results are presented for the linear correlation analysis (R^2^), the mean difference between the HU values in FBP and ASIR reconstructed images and the levels of agreement (± 2 standard deviation) from Bland-Altman analysis. The mean HU values of the images reconstructed by FBP for I = 120 mA are shown for reference.

#### CNR

**Fig 5:**
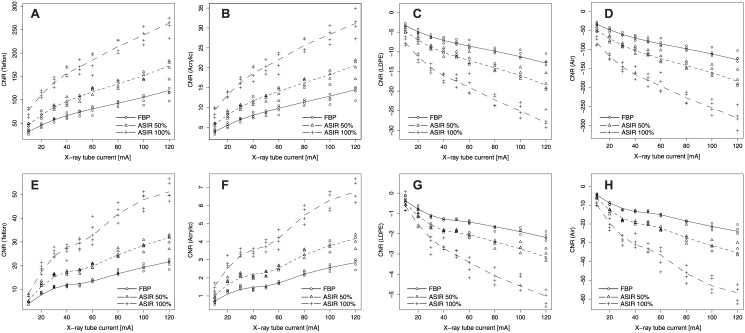
CNR of different sensitometry samples of the Catphan^®^ phantom. CNR of four different sensitometry samples of head geometry (Catphan^®^ phantom, top row A-D) and of the body geometry (Catphan^®^ phantom with additional annulus, bottom row, E-H) for FBP and ASIR reconstructions. The CNRs were examined for Teflon (A, E), Acrylic (B, F), LDPE (C, G), and air (D, H). Due to the definition of the CNR, the slopes of LDPE and air are negative.

The CNR of the head and the body geometry showed different dependencies in relation to the X-ray tube current. The head geometry showed a dependency of |CNR| proportional to √I (R^2^ > 0.94, p < 0.0001, Fig 5A–5D). The CNR of the body geometry showed inflection points for X-ray tube currents in the range of I = 40–60 mA. Therefore, the CNR remained nearly constant for these currents (Fig 5E–5H).

The CNR of the different sensitometry samples in images reconstructed by ASIR50% and ASIR100% were linearly correlated to the CNR of images reconstructed by FBP for both geometries (R_Head_^2^ ≥ 0.97 and R_Body_^2^ ≥ 0.94, both p < 0.0001). The CNRs of the sensitometry samples in the head geometry were higher than the CNRs in the body geometry for equal tube currents and reconstruction parameters (p < 0.0001). For equal X-ray tube currents (equal CT exposures) ASIR50% increased the CNR in the head geometry for the different sensitometry samples by a factor of 1.43 (range: 1.42–1.45) and ASIR100% by a factor of 2.19 (range: 2.15–2.32) compared to images reconstructed by FBP. For the body geometry the CNR was increased by a factor of 1.48 (range: 1.46–1.49) for ASIR50% and 2.54 (range: 2.51–2.57) for ASIR100%.

For the head geometry the CNR of images scanned with I = 30 mA and reconstructed by ASIR50% were not significantly different from the CNR of images scanned with I = 60–80 mA and reconstructed by FBP (p ≥ 0.37). Images scanned with I = 30 mA and reconstructed by ASIR100% showed a significantly better CNR (p < 0.001) than did images reconstructed by FBP with I = 120 mA (Fig 5A–5C). For the body geometry the CNR of images scanned with I = 30 mA and reconstructed by ASIR50% or ASIR100% were not significantly different (p ≥ 0.29) from the CNR of images reconstructed by FBP with I = 80 mA (Fig 5D–5F).

### SPECT Imaging

#### HU Bias, μ Map Bias

CT scans of the SPECT phantom (with and without extension) were performed for different X-ray tube currents. Subsequently, the effect of a possible HU bias was examined for the μ maps and for the reconstructed counts of the SPECT images.

**Fig 6:**
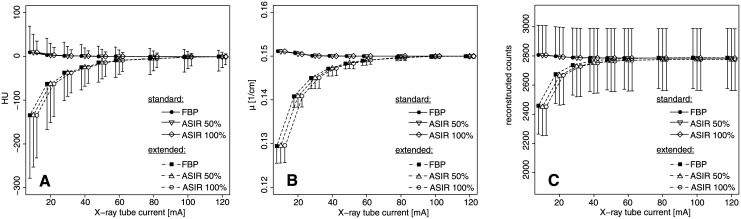
Bias in SPECT-CT examinations. (A) Reconstructed HU values of CT scans reconstructed with FBP and ASIR, (B) calculated attenuation coefficients μ and (C) reconstructed SPECT counts (C) versus X-ray tube current for both SPECT phantoms (standard and extended) and for a fixed tube voltage of U = 120 kVp. The reconstructed SPECT counts (C) of the extended phantom for I = 120 mA are scaled to match with the mean values of the standard phantom for the identic X-ray tube current.

In the case of no extension (standard geometry, Fig 6), the X-ray tube current had a significant effect on the HU values and the μ maps for images reconstructed by FBP (both p < 0.0001, Fig 6A and 6B). The mean of the HU values changed significantly from -0.88 ± 6.63 at I = 120 mA to 9.51 ± 25.51 at I = 10 mA (ΔHU = 10.39 ± 26.71, p < 0.0001). The corresponding μ value changed slightly by 0.73% (Δμ = 0.0011 ± 0.0002 cm^-1^, p < 0.0001). The different ASIR levels had no significant influence on the mean HU value (p > 0.804) or the mean μ (p > 0.768). However, the different x-ray tube currents did not influence the CTAC reconstructed count density inside the standard SPECT phantom (p = 0.37, Fig 6C). Furthermore, the CTAC reconstructed SPECT counts were not influenced by substituting CT data reconstructed by ASIR for the standard CT data reconstructed by FBP (ASIR50%: p = 0.933; ASIR100%; p = 0.738).

The extended SPECT phantom showed a significant influence of the X-ray tube current on the mean HU values, the mean μ and the mean reconstructed SPECT counts (all p < 0.0001, Fig 6A–6C). The estimated μ values were significantly decreased for X-ray tube currents of I ≤ 50 mA compared to I = 120 mA (p < 0.0002). Additionally, the bias in μ values was propagated to the CTAC reconstructed SPECT counts. SPECT counts reconstructed with μ maps calculated from CT scans measured with I ≤ 20 mA differed significantly from SPECT data reconstructed with a μ map measured with I = 120 mA (p ≤ 0.001, Fig 6C). There was a decrease of 11.6 ± 1.8% (p < 0.0001) in the mean counts when the X-ray tube current changed from I = 120 mA to I = 10 mA. For the extended SPECT phantom, the mean HU values of the ASIR50% and the ASIR100% data did not differ significantly from the FBP data (p > 0.864).

Additionally, there was an offset in the reconstructed counts between both phantom geometries (data not shown). The SPECT counts reconstructed with μ maps from CT scans with I = 120 mA were significantly increased by 10.7% in the extended geometry compared to the standard geometry (p < 0.0001).

### Radiation Dose

There was a correlation between the measured CTDI_vol_ and the dose report for both CTDI_vol_-geometries. The scan protocols examined yielded CTDI_vol_-values ranging from 3.70–42.00 mGy (head geometry) and 1.87–20.77 mGy (body geometry). The correlations of the automatically documented and measured values for the CTDI_vol_ head and for the CTDI_vol_ body geometry were CTDI_vol, measured_ = -0.184 + 1.013 × CTDI_vol, documented_ (R^2^ = 1, p <0.0001) and CTDI_vol, measured_ = -0.115 + 1.034 × CTDI_vol, documented_ (R^2^ = 0.99, p < 0.0001), respectively. The results were documented for the different X-ray tube currents and for both CT phantom geometries (Table 2). The calculated effective dose from the CT scan of a hybrid SPECT-CT application was documented for normal male and female patients (Table 2).

**Table 2:** CT exposure for the observed imaging protocol.

	CTDI-Head Geometry		CTDI-Body Geometry		Dose Exposure
Tube current	CTDI_vol,measured_ (CTDI_vol, CT_)	Deviation	CTDI_vol,measured_ (CTDI_vol, CT_)	Deviation	Body-Geometry
					male / female
[mA]	[mGy]	[%]	[mGy]	[%]	[mSv]
10	3.70 (3.53)	+ 4.6	1.87 (1.78)	+ 4.6	0.4 / 0.4
40	14.07 (14.12)	- 0.4	6.93 (7.12)	- 2.7	1.5 / 1.7
80	28.03 (28.25)	- 0.8	13.93 (14.24)	- 2.2	3.0 / 3.5
120	42.00 (42.37)	- 0.9	20.77 (21.36)	- 2.7	4.5 / 5.2

Measured CT exposure (CTDI_vol, measured_), CT exposure automatically documented by the CT scanner (CTDI_vol, CT_) and the percentage deviation between CTDI_vol, CT_ and CTDI_vol, measured_ for the observed LD-CT protocol. Values were documented for the head and body geometry. The effective CT dose for a single abdominal bed position was calculated by the CT-Expo software.

## Discussion

In this paper we analyzed combinations of dose-reduced CT imaging protocols and image reconstruction algorithms with iterative noise suppression to obtain improved LD-CT images for anatomical localization and attenuation correction in hybrid SPECT-CT. We also examined the influence of decreased X-ray tube current on the reconstructed HU values. In diagnostic CT imaging a CT bias in HU values is not considered. However, for LD-CT imaging it is very important to describe effects of a potential bias in HU values concerning μ maps and attenuation correction of emission data.

Using the iterative reconstruction algorithm ASIR for LD-CT imaging, the images were less noisy compared to FBP reconstructions with identical X-ray tube currents. ASIR reduced image noise significantly and provided a noise level that was comparable to FBP reconstructions performed with higher X-ray tube currents. The magnitude of image noise reduction depended on the phantom geometry (attenuation, scatter, etc.) and the parameterization of the iterative reconstruction algorithm. The most distinct improvement with respect to image noise was achieved with ASIR100%. Through noise reduction, ASIR affected the estimated CNR too. The Catphan^®^ phantom (with and without annulus) showed the expected dependency |CNR| ~ √I for all the algorithms. Decreasing X-ray tube current in combination with ASIR reduced the CT-related radiation exposure, preserved or even reduced the noise level, and increased the CNR.

The images reconstructed by ASIR and by FBP showed no differences with respect to the HU values for the Catphan^®^ phantom (with and without annulus) for identical X-ray tube currents and the different material modules were well correlated for both types of reconstruction. However, there was a significant deviation in HU values (HU bias) for low X-ray tube currents (I ≤ 60 mA) for the Catphan^®^ phantom extended by the additional annulus, representing the abdominal region of a standard patient, compared to scans performed with higher X-ray tube currents. The HU bias observed for low X-ray tube currents and the artificial influence of the CTAC on the emission data was simulated by Xia et al. [10] for a PET-CT system. In our experimental examinations the HU bias already occurred at higher X-ray tube currents than those simulated by Xia et al. (I = 5–10 mA, U = 120 kVp, (11)). This deviation might be attributable to differences in the examined/simulated phantom geometry and the theoretical model used for the scanner vs. the CT employed (e.g. CT detector, electronic circuits, etc.). Finally, electronic noise and correction for the mean dark current of the system were discussed as possible causes of the HU bias [27–29].

The pure CT bias of a hybrid PET-CT system was observed by different authors [19]. More recently, the HU bias has been reproduced by phantom examinations for a PET-CT by Abella et al. [30]. Furthermore, the HU bias effect was not observed in general [18]. In addition, experience gathered with LD-CT imaging apart from hybrid applications (e.g. CCTA) have been reported [15,16]. But in this field of application imaging conditions are significantly different, so that visualization of the structures of interest (e.g. arteries) are improved by the use of an additional contrast medium. Furthermore, the influence of image degrading effects (e.g. attenuation) are significantly reduced in the thoracic region compared to the abdominal region. Our set-up (abdominal region) was performed under considerably different imaging conditions, with a different focus of optimization.

The influence of the HU bias on hybrid SPECT imaging was examined with an elliptical SPECT phantom that represents a standard geometry in nuclear medicine. It is noteworthy that we did not receive functional data (SPECT data) from the CT phantom (Catphan^®^ phantom). Investigations with the sole elliptical SPECT phantom (standard patient geometry) revealed a slight HU bias and therefore only a small bias within the μ map. The reconstructed SPECT counts were not affected. This would also hold true for other regions of a standard patient with decreased attenuation (e.g. thorax, head). However, investigations with an extended version of the SPECT phantom (extended geometry), surrounded with 13 water-filled plastic bottles, which is somewhat closer to abdomen of an obese patient with increased attenuation, showed a significant bias in the HU values with decreasing X-ray tube current. The induced biases within the μ map and the reconstructed SPECT images were detected for I ≤ 50 mA (μ values) and for I ≤ 20 mA (reconstructed SPECT counts). Here, the reduction of CT exposure was limited by the bias effect in the HU values and its influence on the reconstructed SPECT information propagated by CTAC. Due to the fact that HU bias depends on the phantom geometry, the characteristic of the imaging system and the implementation of calculating μ maps from HU values, no general effect can be postulated. As a result, the HU bias should be considered as a property of the imaging system (e.g. hardware, reconstruction algorithm). Several authors have proposed new data processing technologies to overcome the influence of HU bias in LD-CT for hybrid imaging [27,31]. A first implementation has recently become available for current hybrid PET-CT and SPECT-CT devices (Q.AC, GE Healthcare, Milwaukee, USA).

However, our study also underlines the need for complementary CT and SPECT phantoms. The standard phantoms for CT and SPECT imaging do not match perfectly (e.g. different dimensions, different composition of materials) and this hampers the optimization process for hybrid imaging systems. In our experiments the standard SPECT phantom (cross-section about 517 cm^2^) did not show a HU bias effect whereas the Catphan^®^ phantom with additional annulus (cross-section 952 cm^2^) showed the HU bias effect discussed!

Finally, the decrease in X-ray tube current was only one parameter for dose optimization. Decreasing the CT tube current reduces the number of X-ray photons, which leads to a linear decrease in radiation dose for a given acquisition time within a given scan protocol. An alternative for the optimization of radiation dose is the X-ray tube voltage. Decreasing the X-ray tube voltage lowers the hardness of the X-ray spectra and that leads to a decrease in photon penetration capacity applicable to small patients (i.e. children). The scan protocol, especially the tube voltage settings, was chosen following the strategies in diagnostic imaging. Besides, acquisitions with lower tube voltages (80 kVp, 100 kVp) were performed outside the study. But those results were not analyzed in the context of hybrid imaging with LD-CT of adult patients because of the highly reduced image quality. This effect is known from diagnostic CT imaging [32].

In future studies, the influence of the different ASIR-levels should be analyzed in more detail in phantom and patient set-ups to get an optimal dose reduction with respect to image quality for LD-CT applications (e.g. localization diagnostics, attenuation correction) in hybrid imaging. The iterative reconstruction algorithm examined (ASIR) was the first implementation of advanced CT reconstruction algorithms available in hybrid SPECT-CT imaging. Currently, novel implementations are coming up with a comprehensive modeling of image geometry (e.g. the physical dimension of the focal spot) and detector elements [33]. Studies of diagnostic CT imaging have reported a decrease in CT exposure (up to -75%) for the application of this refined algorithm [34], so that the use of that iterative reconstruction algorithm can further improve LD-CT in hybrid imaging devices.

## Conclusion

We have presented a systematic study for the use of an iterative image reconstruction algorithm (ASIR) for LD-CT that is widely used in diagnostic CT imaging. The algorithm was tested with regard to image quality and radiation dose management in LD-CT hybrid imaging by phantom examinations. The advantages of ASIR in LD-CT imaging were the reduction of image noise and the increase in the CNR compared to FBP reconstructions of the same CT scan. For that reason, the application of ASIR provides an opportunity to reduce CT radiation exposure without a significant impairment of image quality. However, there is a systematic limitation for CT dose reduction in hybrid SPECT-CT imaging for both reconstruction algorithms. A HU bias was observed in examinations of the extended phantom geometries (obese patients) for very low X-ray tube currents (I ≤ 20mA). The HU bias was propagated to the reconstructed SPECT counts by CTAC.

Additionally, we have underlined the need for complementary CT and SPECT phantoms.
